# The Sensory Abnormalities and Neuropsychopathology of Autism and Anxiety

**DOI:** 10.7759/cureus.8071

**Published:** 2020-05-12

**Authors:** Sirisha K Gara, Ashok G Chhetri, Montaser Alrjoob, Sassi Ashraf Ali Abbasi, Ian H Rutkofsky

**Affiliations:** 1 Primary Care, California Institute of Behavioral Neurosciences and Psychology, Fairfield, USA; 2 Primary Care, Department of Research, California Institute of Behavioral Neurosciences and Psychology, Fairfield, USA; 3 Internal Medicine, California Institute of Behavioral Neurosciences and Psychology, Fairfield, USA; 4 Psychiatry, California Institute of Behavioral Neurosciences and Psychology, Fairfield, USA

**Keywords:** anxiety disorder, autism spectrum disorder, autism spectrum disorder and anxiety disorder, sensory in autism and anxiety, autism spectrum disorder and emotion, anxiety and autism linked to emotion, autistic disorder and anxiety connected to psychopathology

## Abstract

Autism spectrum disorder (ASD) is a developmental disorder of interpersonal communications and restricted interest and deficits in sensory and social interactions. It co-occurs with anxiety and mostly in 30% of cases related to specific phobia. This review article summarises the sensory association between anxiety and ASD. The role of emotions and neurobiology discussed and sensory over-reactivity (SOR) was related to ASD and anxiety. PubMed database systematically searched for related articles on ASD and anxiety. The keywords used are autism spectrum disorder, autism spectrum disorder and emotion, anxiety disorder, sensory in autism and anxiety, and psychopathology. The results were most significant and related to the sensory association between ASD and anxiety. Out of 19 studies discussed, there were eight systematic reviews with meta-analysis, seven systematic reviews, three traditional reviews, and one included both systematic reviews with randomized controlled trials (RCTs). However, due to possible limitations and considerations, like small sample size and few clinical trials; hence, further recommendations to randomized clinical trials and cohort studies warranted. This review article helps scientists to plan and focus on necessary studies and possible screening for the disease to improve possible clinical outcomes. People gain awareness of the disease. Early recognition, as well as educational, behavioral, and family therapy, might decrease symptoms and support learning and development in children.

## Introduction and background

Autism spectrum disorder (ASD) is a neurological disease characterized by deficits in social communication as well as repetitive behaviors and restricted interests. ASD is a neurodevelopmental disorder with increased prevalence in children and adults over the past few decades. Specific phobia is associated with ASD in 30% cases and includes obsessive-compulsive disorder 17%, social anxiety disorder, and agoraphobia 17%, generalized anxiety disorder 15%, separation anxiety disorder 9%, and panic disorder 2%. Based on parent analysis, 25.2% of boys and 19.5% of girls with ASD are out of margin for generalized anxiety disorder (teacher report: 23.3% and 20.8% for boys and girls, respectively). For separation anxiety, 6.7% of boys and 7.1% of girls were above the screening threshold via parent-report (teacher report: 13.8% and 8.0%, respectively) [1-3].

Severe signs and symptoms of anxiousness frequently co-occur in ASD [4,5]. Nearly 40% of human beings had elevated levels of anxiousness, and it is related to comorbidity in both children and adults. The anxiousness determined right here is regular with previous evaluations of the ASD literature. In addition to the core signs and symptoms of ASD; comorbid psychiatric prerequisites are highly prevalent, increasing impairment, and complicating prognosis and treatment [6-8]. There is widespread evidence that people with ASD are at heightened danger for anxiety and anxiety disorders, which can propose chronic distress, exacerbate ASD symptoms, and amplify behavioral troubles. The sensory feature is associated with repeated touching or painful response to objects. Both extremes may show up in identical children [9-13].

This review mostly focuses on the sensory association between ASD and anxiety however conjointly discusses three ideas for understanding the event and maintenance of anxiety in ASD: (1) atypical sensory function, that is often enclosed within the diagnostic criteria for ASD; (2) problem identifying/labeling emotions (alexithymia), that has shown to be severe in ASD; and (3) neurobiology connected to anxiety and ASD. Whereas these ideas come back from completely different backgrounds, existing analysis shows that they are closely related. A significant challenge is to outline how these ideas diverge biologically. Improved understanding of the psychological feature and emotional mechanisms that underlie anxiety in ASD could give insight relating to the psychopathology and psychophysiology of each condition and make additional specific targets for biological and behavioral intervention [14-16].

**Figure 1:**
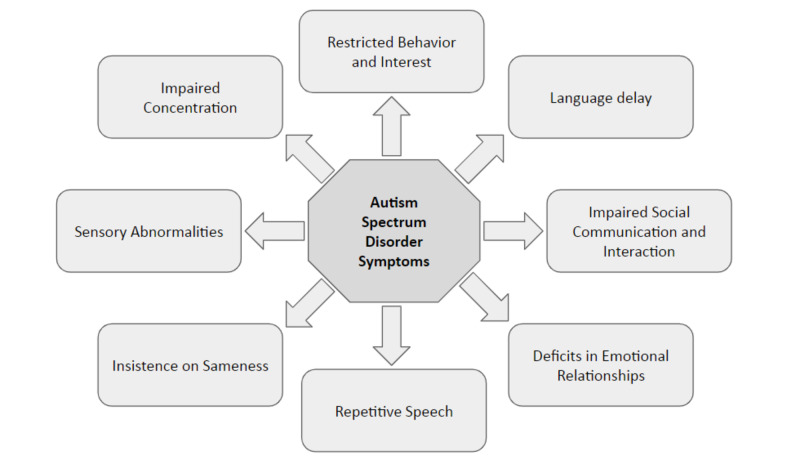
ASD symptoms ASD, autism spectrum disorder

After understanding the ideas and prevalence of ASDs and anxiety, we believe that there is a need to evaluate the link between ASD and anxiety and the way they are connected. Because of the increasing prevalence of tension in ASDs, there is a necessity to grasp the factors taking part in a task for any management and treatment. This review aims to summarize the recent literature related to abnormalities in sensory functioning in people with ASD, together with proof linked to the neurobiology of those symptoms, their clinical correlation, and their treatment. Abnormalities in responses to sensory stimuli are incredibly prevalent in people with ASD. The underlying neurobiology of those symptoms is unclear, however many theories projected linking potential etiologies of neural pathology with well-known abnormalities in brain structure and performance that are related to ASD [17]. The following symptoms characterize autism spectrum disorder (Figure 1).

## Review

Methods and results

PubMed database systematically searched using the keywords anxiety disorder, autism spectrum disorder, autism spectrum disorder and anxiety disorder, sensory in autism and anxiety, autism spectrum disorder and emotion, anxiety, and autism linked to emotion. After a search with the exclusion and inclusion criteria applied to full text and articles reviewed within the last 10 years yielded the following results: anxiety disorder 60537, autism spectrum disorder 22408, autism spectrum disorder and anxiety disorder 1572, sensory in autism and anxiety 160, anxiety and autism linked to emotion 70. Combined mesh keywords used narrowed the search to the results below. Autistic disorder and anxiety 132, autistic disorder, and anxiety connected with psychopathology 40.

All the duplicate and impertinent articles were excluded from the study. All types of clinical studies included without limiting to a specific category. The reviews restricted within the last 10 years. Google Scholar has conjointly been used as a reference wherever applicable, along with PubMed. The following studies are related to the sensory association between autism and anxiety (Table 1 ).

**Table 1:** Results References listed in the table also includes [30,31,33,38] OCD, obsessive compulsive disorder; ASD, autism spectrum disorder; GAD, generalised anxiety disorder; ANOVA, analysis of variance; EC, effortful control; PIP, peer interaction paradigm; SOR, sensory over-reactivity Anxiety scales ADOS, Autism Diagnostic Observation Schedule; MSEL, Mullen Scales of Early Learning; ITSEA, Infant Toddler Social Emotional Assessment; BAI, Beck Anxiety Index; CES-D, Center for Epidemiological Studies Depression Inventory; PSI, Parenting Stress Index; FLIS, Family Life Impairment Scale; CASI, Children and Adolescent Symptom Inventory; MASC, Multidimensional Anxiety Scales for Children; ADIS, Anxiety Diagnostic Interview Scale; PARS, Pediatric Anxiety Rating Scale; ADAMS, Anxiety Depression and Mood Scale; RCADS, Revised Child Anxiety and Depression Scale; SCARED, Screen for Child Anxiety Related Disorders; RCAMS, Revised Children's Manifest Anxiety Scales

#	Author (Year)	Type of study	Country	Study’s focus	Findings	Summary
1	White et al. (2009) [6]	Systematic review	USA	Autism Anxiety	A systematic review of 40 papers.	Prevalence, treatment, and methodology.
2	Green and Ben-Sasson (2010) [30]	Review	USA	Anxiety Sensory over-reactivity	Theoretical models between SOR and anxiety.	A strong association between SOR and anxiety.
3	van Steensel et al. (2011) [3]	Systematic review and meta-analysis	Netherlands	Anxiety and ASDs	Identified 31 studies with 2121 people and conducted standard questionnaires or diagnostic interviews, phobia (29.8%) followed by OCD (17.4%) and social anxiety disorder (16.6%).	The link between the specific anxiety disorder and ASD subtype has been identified.
4	Green et al. (2012) [33]	Systematic review with meta-analysis	USA	Anxiety Sensory over-reactivity in ASD in toddlers	n = 149 toddlers. Paired t sample test, t(148) = −3.00, p = .003 but mean SOR scores were stable across time, t(148) = −1.10, p = 0.275.	SOR predicts a change in anxiety across toddlerhood.
5	Green et al. (2013) [31]	Systematic review	USA	ASD, sensory stimuli	n = 25 high functioning youth age 25 assessed aversive auditory and visual stimuli during a functional MRI scan.	ASD associated with increased activation of the hippocampus, amygdala, and orbitofrontal cortex.
6	Ben-Sasson et al. (2013) [18]	Systematic review with meta-analysis	Israel	ASD SOR Stress in Toddlers	n = 175. ADOS, MSEL composite score, ISTEA anxiety symptoms BAI, CES-D at time 1 explained 39-45% of the variance.FLIS and PSI did not change over time.	SOR is related to stress.
7	Case-Smith (2015) [19]	Systematic review	USA	Autism and sensory processing	Two randomized controlled trials and 19 studies reviewed and 15 were related to therapy.	Sensory integration therapy reduced sensory problems.
8	Sullivan et al. (2014) [20]	Systematic review	USA	Anxiety Migraine Sensory Over-reactivity	81 children aged 7-17 years. ASD and migraines p<0.01 sensory hyperreactivity and anxiety ρ = 0.31, p = 0.005.	Greater SOR with ASD and migraines.
9	Kerns et al. (2014) [5]	Systematic review and meta-analysis	USA	Anxiety and autism spectrum disorders in youth	n = 59, 7-17 years, Q > 60 with ASD conducted semi-structured interviews and parent reports. 17% traditional anxiety, 15% atypical, and 31% both.	Supported the association between Anxiety and ASD.
10	Green et al. (2015) [38]	Systematic review	USA	ASD Sensory over-reactivity	Mean age 14 years. n = 38. Functional MRI imaging was done. Participants with ASD have a stronger activation of the amygdala p < 0.05. ASD with the SOR subgroup showed decreased neural habituation than without SOR. z > 1.70, p < 0 .05.	Youth with ASD show sensory limbic hyperreactivity through prefrontal downregulation of the amygdala.
11	Lecavalier et al. (2014) [11]	A systematic review and clinical trials	USA	Anxiety ASD	Over a 14-month period, monthly conferences were held and two face-to-face meetings were scheduled. 38 published studies, 10 assessment measures were reviewed and four were appropriate for clinical trials. Anxiety scales: ADAMS - 28-point scale, RCADS - 47-item scale, SCARED - 41-item scale.CASI, MASC, ADIS, PARS are 20-point scales useful for more clinical trials. RCAMS is used for depression and anxiety.	Anxiety in ASD needs for treatment.
12	Neil et al. (2016) [21]	Systematic review with meta-analysis	UK	Anxiety and sensory over-reactivity in children	64 autistic and 85 typically developing children aged 6–14 years. Intolerance of uncertainty role in ASD.	Both children with and without autism associated with it.
13	Corbett et al. (2016) [22]	Systematic review with meta-analysis	USA	ASD, sensory sensitivity	n = 80, Children between 8 and 12 years. ASD and stress with cortisol levels assessed by T test and ANOVA. PIP [F (1, 77) = 5.77, p = 0.02]. P (r = -0.242, p = 0.03), and positively correlated with SSS (r = 0.273, p = 0.02), P (p < 0.05).	Stress relation to SOR.
14	Kerns et al. (2017) [23]	Systematic review with meta-analysis	USA	Anxiety ASD	Sample of 69 youth with ASD, comorbid symptoms 0.85-0.98, κ = 0.67-0.91, anxiety like symptoms 0.87-0.95, κ = 0.77-0.90.	Results conclude the ADIS/ASA measures anxiety in youth with ASD.
15	Uljarević et al. (2017) [24]	Systematic review with meta-analysis	AUS	ASD, anxiety sameness	Study between 71-year-old adolescents and younger individuals with n=71. IS associated with EC (r = -0.39, p = 0.001) and anxiety (r = 0.45, p < 0.001).and anxiety was in turn associated with EC (r = 0.44, p < 0.001).	First revealed the relation between ASD and anxiety.
16	South and Rodgers {2017) [25]	Systematic review	USA	Autism Anxiety Sensory, emotions	Intolerance of uncertainty, Autism, Anxiety and emotions (n = 76).	Associations and the need for the investigation.
17	Postorino et al. (2017) [26]	Review	USA	Anxiety ASD OCD	Anxiety and OCD prevalence in ASD.	Higher correlation.
18	Hwang et al. (2019) [27]	Systematic review	AUS	Anxiety and sensory sensitivity	A sample size of 176 individuals with a mean age 42 included in the study.	Intolerance of uncertainty in anxiety and SOR.
19	Bitsika et al. (2019) [28]	Systematic review	AUS	GAD ASD Sensory features	Test conducted on a small sample size of 150 males limited to age 6 to 18 years.	Higher association between ASD and GAD than sensory.

Discussion

**Figure 2:**
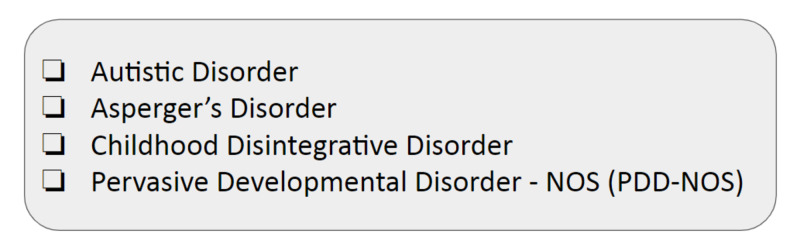
PDD types PDD, pervasive developmental disorder; NOS, not otherwise specified

According to the Diagnostic and Statistical Manual of Mental Disorders, 5th edition: DSM-5 criteria, ASD is a type of pervasive developmental disorders (PDDs). PDD is classified into four categories, as shown below. ASD is of increased prevalence 20/10,000, and PDD-NOS is 30/10,000, Asperger's and childhood disintegrative disorder are rare about 2/10,000 (Figure 2).

 

Sensory Role in Autism and Anxiety

Anxiety issues lead to hyperarousal, hypervigilance, and bias, coupled with over-reactivity in ASDs. Hypervigilance recognized as a problem in risk-based emotional regulation. Signs and symptoms of hyperarousal in the control of emotions causes sensory over-reactivity (SOR). These signs and symptoms of anxiousness cause learning that is preservation and exacerbation of the cycle between anxiousness and SOR. It is the necessary model of nervousness that explains the SOR. Anxiety and associated SOR though they are not causally connected maintained by the treatment of an unknown variable dominant link that’s via the amygdala that may be a neurohormonal correlation, whenever there’s an associate activity of amygdala this nervousness, and SOR happens in people. Over responsivity to sensory stimuli may be a common symptom of the amygdala. ASDs are understudied, due to the reality it had been once only recently delivered to DSM-5 diagnostic criteria. Neural and behavioral over-reactivity associated with each sensory and emotional area in early existence with ASD. The atypical sensory process has been represented in the autism spectrum altogether, and there has been a tested relationship between sensory process, adaptive behaviors, and interest capabilities in teenagers with ASD. There was a vast influence on of these adaptive conduct and activity competencies on ASD. SOR may be a good predictor of the conventional behaviors in ASD and changes in anxiety [29-33].

Sensory Over-Reactivity

SOR is associated with gastrointestinal (GI) problems in teenagers with anxiety and ASD. Children with predominant symptoms of SOR are related to persistent GI troubles like pain abdomen, diarrhea, and bloating. Children with different kinds of GI issues were related to nervousness and SOR. Studying this sensory function has also led the researchers to discover an association with Williams syndrome (WS) and migraines. ASD and WS share common psychopathology touching on to sensory processing and repetitive behaviors. In ASD and WS, difficulties processing the sensory factors of the environment, repetitive behaviors, and high degrees of anxiety co-occur. Direct significant relationships between sensory elements and repetitive behaviors were observed solely for the ASD group. Sensory difficulties maintained by linear relationships related to repetitive behaviors in kids with ASD. In WS, the relationship is mediated via intolerance of uncertainty. The findings support that there is a complexity of the mechanisms underlying the link between sensory processing and repetitive behaviors across neurodevelopmental issues. Children with one type of GI problem had higher levels of anxiety and SOR. GI troubles and anxiety were strongly linked [34,35].

Role of Emotions in ASD and Anxiety

Alexithymia is related to a decreased interest to spot and describe one’s feelings, which leads to reduced sympathy and an impaired ability to acknowledge the emotions. The degree of alexithymia predicted the eye fixation. In addition to social impairment, an emotional disturbance related to ASD. The anterior insula regulates the alexithymia, and the strength of the response is lesser in ASD, and there was a variation from healthy individuals. Empathy deficits in autism are related to emotions and not a feature of impaired social communications. The GABA and glutamate are the neurotransmitters involved in the neurohormonal mechanisms [36-38].

Neurobiology in Anxiety and ASD

In ASD, there has been a greater activation in the fundamental sensory areas like auditory and visual cortices and thalamus as in emotion processing regions like the amygdala, hippocampus, and prefrontal cortex. The thalamic response and amygdala influenced the higher cortical areas of anxiety. The amygdala sends the signal to the hippocampus and maintains conditioning and fear. The amygdala and the red nucleus of Stria Terminalis implicated in the characteristics of anxiety disorders. The amygdala and the red nucleus of the Stria Terminalis involved in the symptoms of anxiety disorders. Somatostatin-expressing neurons in the central amygdala (CEA) controls anxiousness through modulation of Stria Terminalis, a technique that mediated with the aid of dynorphin signaling in the CEA [39].

Therapy in Anxiety and ASD

The sensory association also helped in targeting cognitive behavioral therapy, which has proven to be effective in patients with anxiety and ASD. The cognitive-behavioral treatment helps children with ASD and anxiety, which includes psychoeducation, cognitive restructuring, self-talk, relaxation, and exposure to feared stimuli. A manualized CBT intervention has enhanced for the children with ASD by addressing adaptive skills and poor social skills in addition to the core anxiety problems. Psychopharmacological treatments with selective serotonin reuptake inhibitors have been effective in treating patients with anxiety. Autism is mostly associated with sensory and self-regulatory processes as controls, and massage therapy also played a beneficial role in managing the sensory symptoms in patients with anxiety. Early identification of the disease might improve the outcome in language, cognitive, and adaptive skills; hence, we have to develop a new screening tool for the disease [40-43]. Studying the relationship between SOR and anxiety in individuals with ASD by this diagnostic overlap helps in making the occupational therapists proceed with the treatment benefits with accuracy.

## Conclusions

This review article's primary focus is the sensory association between ASD and anxiety and also the associated neuropathology. Once the association between ASD and anxiety established, it is essential to search out out if, to any extent, further factors are playing a role because it could be a leading cause and most outgrowing in youngsters. The main focus was on sensory association and also the emotional performance that helped in treating patients with ASD and anxiety. SOR plays an essential part in the discussion. The pathway association with autism and anxiety helped in the strengthening of the relationship between autism and anxiety.

SOR plays a critical role in the debate. This review not only solely summarises all the possible links the sensory, emotional, and neurobiology in autism and anxiety; however, it also reveals the sensory association with Williams syndrome and Gastrointestinal disorders. This analysis helps improve the treatment outcomes among individuals with autism spectrum disorders and anxiety. However, additional clinical trials and cohort studies are necessary to strengthen the effect of the association between them. The new screening tools for further diagnostic purposes can improve clinical outcomes through early treatment and management.
